# The long non-coding RNA HOTAIR indicates a poor prognosis and promotes metastasis in non-small cell lung cancer

**DOI:** 10.1186/1471-2407-13-464

**Published:** 2013-10-08

**Authors:** Xiang-hua Liu, Zhi-li Liu, Ming Sun, Jing Liu, Zhao-xia Wang, Wei De

**Affiliations:** 1 Department of Biochemistry and Molecular Biology, Nanjing Medical University, Nanjing, Jiangsu 210029, People’s Republic of China; 2 Department of Oncology, The Second Affiliated Hospital, Nanjing Medical University, Nanjing, Jiangsu 210011, People’s Republic of China

**Keywords:** Long non-coding RNA, HOTAIR, Non-small cell lung cancer, Prognosis, Metastasis

## Abstract

**Background:**

The identification of cancer-associated long non-coding RNAs and the investigation of their molecular and biological functions are important for understanding the molecular biology and progression of cancer. HOTAIR (*HOX transcript antisense intergenic RNA*) has been implicated in several cancers; however, its role in non-small cell lung cancer (NSCLC) is unknown. The aim of the present study was to examine the expression pattern of HOTAIR in NSCLC and to evaluate its biological role and clinical significance in tumor progression.

**Methods:**

Expression of HOTAIR was analyzed in 42 NSCLC tissues and four NSCLC cell lines by quantitative reverse-transcription polymerase chain reaction (qRT-PCR). Over-expression and RNA interference (RNAi) approaches were used to investigate the biological functions of HOTAIR. The effect of HOTAIR on proliferation was evaluated by MTT and colony formation assays, and cell migration and invasion were evaluated by transwell assays. Tail vein injection of cells was used to study metastasis in nude mice. Protein levels of HOTAIR targets were determined by western blot analysis. Differences between groups were tested for significance using Student’s t-test (two-tailed).

**Results:**

HOTAIR was highly expressed both in NSCLC samples and cell lines compared with corresponding normal counterparts. HOTAIR upregulation was correlated with NSCLC advanced pathological stage and lymph-node metastasis. Moreover, patients with high levels of HOTAIR expression had a relatively poor prognosis. Inhibition of HOTAIR by RNAi decreased the migration and invasion of NSCLC cells *in vitro* and impeded cell metastasis *in vivo*. HOXA5 levels were affected by HOTAIR knockdown or over-expression *in vitro*.

**Conclusions:**

Our findings indicate that HOTAIR is significantly up-regulated in NSCLC tissues, and regulates NSCLC cell invasion and metastasis, partially via the down-regulation of HOXA5. Thus, HOTAIR may represent a new marker of poor prognosis and is a potential therapeutic target for NSCLC intervention.

## Background

Non-small cell lung cancer (NSCLC), including adenocarcinoma and squamous cell carcinoma, is the predominant form of lung cancer, and accounts for the majority of cancer deaths worldwide [1]. Despite recent advances in clinical and experimental oncology, the prognosis of lung cancer is still unfavorable, with a 5 year overall survival rate of only around 11% [2]. Thus, a detailed understanding of the mechanisms underlying NSCLC development and progression are essential for improving the diagnosis, prevention and treatment of this disease. Recently, accumulating evidence has shown that non-coding RNAs (ncRNAs) may be involved in NSCLC pathogenesis, providing new insights into the biology of the disease.

Recent improvements in genome-wide surveys and high throughput transcriptome analysis have revealed that human genome contains only ~20,000 protein-coding genes, representing <2% of the total genome while a substantial fraction of the human genome can be transcripted into many short or long noncoding RNAs with limited or no protein-coding capacity [3,4]. Among the different classes of ncRNAs, microRNAs have received the most attention and have been shown to play many important roles in cancer via post-transcriptional silencing of specific target mRNAs [5,6]. However, one of the emerging areas in ncRNA research is the newly discovered class of long non-coding RNAs (lncRNAs). The participation of lncRNAs in a wide repertoire of biological processes has been intensely researched, because virtually every step of mRNA biology, from transcription to mRNA splicing, RNA decay and translation can be influenced by these molecules [7-9]. Multiple lines of evidence link dysregulation of lncRNAs to diverse human diseases, especially cancers. Several studies suggest that many lncRNAs can be associated with chromatin-modifying complexes to affect epigenetic information and to confer multiple properties that are required for tumor progression and a metastatic phenotype [10-12]. Therefore, identification of cancer-associated lncRNAs and the interplay between lncRNAs and protein-coding genes are important topics in the field of cancer biology, in which lncRNAs may provide a missing piece of the oncogene and tumor suppressor network puzzle.

HOTAIR (*Hox transcript antisense intergenic RNA* ) is one of the few well-documented lncRNAs, with a length of 2158 bp and a functional role in trans-silencing [13]. Recently, HOTAIR has been determined to be a negative prognostic indicator in breast, colon, liver, and pancreatic cancer patient survival, and increased HOTAIR expression in patients has been correlated with enhanced breast and colon cancer metastasis. Meanwhile, HOTAIR knockdown can inhibit cell invasion and proliferation, alter cell cycle progression and induce apoptosis, indicating that HOTAIR can play a direct role in the modulation of cancer progression [14-17]. Nevertheless, little is known about the impact of HOTAIR on NSCLC carcinogenesis or metastasis.

To better understand the role of HOTAIR in NSCLC development and progression, we investigated the expression pattern of HOTAIR in NSCLC tissues and analyzed its relationship to clinical pathological features. We also explored HOTAIR function during NSCLC progression using *in vitro* and *in vivo* assays and investigated the molecular mechanisms by which HOTAIR contributes to the phenotypes of NSCLC cells.

## Methods

### Tissue collection

Forty-two paired NSCLC and adjacent non-tumor lung tissues were obtained from patients who underwent surgery at Jiangsu Province Hospital between 2006 and 2007 and were diagnosed with NSCLC (stage II, III, and IV) based on histopathological evaluation. Clinicopathological characteristics including tumor-node-metastasis (TNM) stage were collected. No local or systemic treatment was conducted in these patients before surgery. All collected tissue samples were immediately snap-frozen in liquid nitrogen and stored at -80°C until use. The study was approved by the Research Ethics Committee of Nanjing Medical University, China. Written informed consent was obtained from all patients.

### Cell lines and culture conditions

Three NSCLC adenocarcinoma cell lines (A549, SPC-A1, NCI-H1975), a NSCLC squamous carcinomas cell line (SK-MES-1), and a normal human bronchial epithelial cell line (16HBE) were purchased from the Institute of Biochemistry and Cell Biology of the Chinese Academy of Sciences (Shanghai, China). Cells were cultured in RPMI 1640 or DMEM (GIBCO-BRL) medium supplemented with 10% fetal bovine serum (FBS), 100 U/ml penicillin and 100 mg/ml streptomycin (Invitrogen, Carlsbad, CA, USA) in humidified air with 5% CO_2_ at 37°C.

### RNA extraction and quantitative real-time PCR

Total RNA was extracted from tissues or cultured cells with TRIzol reagent (Invitrogen) according to the manufacturer’s protocol. qRT-PCR assays were performed to detect HOTAIR expression using the PrimeScript RT reagent Kit and SYBR Premix Ex Taq (TaKaRa, Dalian, China) according to the manufacturer’s instructions. Results were normalized to the expression of glyceraldehyde-3-phosphate dehydrogenase (GAPDH). The primers used were as follows: HOTAIR sense, 5’-CAGTGGGGAACTCTGACTCG-3′ and antisense, 5′-GTGCCTGGTGCTCTCTTACC-3′; GAPDH sense, 5′-GGGA GCCAAAAGGGTCAT-3′ and antisense, 5′-GAGTCCTTCCACGATACCAA-3′. qRT-PCR and data collection were performed on an ABI 7500. qRT-PCR results were analyzed and expressed relative to CT (threshold cycle) values, and then converted to fold changes.

### Plasmid construction

To generate a HOTAIR expression vector, we amplified a full-length HOTAIR fragment by PCR from SPC-A1 cDNA. Oligonucleotides for amplification of HOTAIR (sense, 5′-CATGGATCCACATTCTGCCCTGA TTTCCGGAACC-3′ and antisense, 5′-ACTCTCGAGCCACCACACACACACA ACCTACAC-3′) were designed to incorporate external *HindIII* and *XhoI* sites, respectively. The PCR product was verified and subcloned into the mammalian expression vector pCDNA3.1 (Invitrogen).

### Cell transfection

Plasmid vectors (pCDNA3.1-HOTAIR and pCDNA3.1-NC) for transfection were prepared using DNA Midiprep or Midiprep kits (Qiagen, Hilden, Germany). Three individual small interfering RNA (siRNAs) and scrambled negative control siRNA (si-NC) were purchased from Invitrogen (Invitrogen). The target sequences for HOTAIR siRNAs were as follows: (si-HOTAIR1, 5′-AAAUCCAGAACCCUCUGACAUUUGC-3′, si-HOTAIR2, 5′-UUAAGUCUA GGAAUCAGCACGAAGC-3′ and si-HOTAIR3, 5′-CAUAUUAUAGAGUUGCU CUGUGCUG-3′. pCDNA3.1-HOTAIR or pCDNA3.1-NC was transfected into cultured A549 cells, and HOTAIR siRNAs or si-NC were transfected into cultured SPC-A1 cells. A549 and SPC-A1 cells were grown on six-well plates to confluency and transfected using Lipofectamine 2000 (Invitrogen) according to the manufacturer’s instructions. Forty-eight hours after transfection, cells were harvested for qRT-PCR or western blot analyses.

### Cell proliferation assays

Cell proliferation was monitored using Cell Proliferation Reagent Kit I (MTT) (Roche Applied Science). Si-HOTAIR-transfected SPC-A1 cells (3000/well), and pCDNA3.1-HOTAIR-transfected A549 cells (2000/well) were allowed to grow in 96-well plates. Cell proliferation was documented every 24 h following the manufacturer’s protocol. All experiments were performed in quadruplicate. For the colony formation assay, a total of 500 HOTAIR siRNA-transfected SPC-A1, or pCDNA3.1-HOTAIR-transfected A549 cells were placed in a fresh six-well plate and maintained in media containing 10% FBS, replacing the medium every 4 days. After 14 days, cells were fixed with methanol and stained with 0.1% crystal violet (Sigma-Aldrich). Visible colonies were manually counted. For each treatment group wells were assessed in triplicate.

### Flow-cytometric analysis of apoptosis

SPC-A1 cells, transiently transfected with si-HOTAIR or si-NC, were harvested 48 h after transfection by trypsinization. After double staining with FITC-Annexin V and propidium iodide, cells were analyzed by flow cytometry (FACScan; BD Biosciences) using CellQuest software (BD Biosciences). Cells were discriminated into viable cells, dead cells, early apoptotic cells, and apoptotic cells, and then the relative ratio of early apoptotic cells was compared to the control from each experiment. All samples were assayed in triplicate.

### Cell migration and invasion assays

For the migration assays, 48 h after transfection, 5 × 10^4^ cells in serum-free media were placed into the upper chamber of an insert (8 μm pore size, Millipore). For the invasion assays, 1 × 10^5^ cells in serum-free medium were placed into the upper chamber of an insert coated with Matrigel (Sigma-Aldrich). Media containing 10% FBS were added to the lower chamber. After incubation for 24 hours, the cells remaining on the upper membrane were removed with cotton wool, whereas the cells that had migrated or invaded through the membrane were stained with methanol and 0.1% crystal violet, imaged, and counted using an IX71 inverted microscope (Olympus, Tokyo, Japan). Experiments were independently repeated three times.

### Western blot analysis and antibodies

Cells were lysed using RIPA protein extraction reagent (Beyotime, Beijing, China) supplemented with a protease inhibitor cocktail (Roche, CA, USA) and phenylmethylsulfonyl fluoride (Roche). Protein concentration was measured using the Bio-Rad protein assay kit. Approximately 50 μg of protein extract was separated by 10% SDS-polyacrylamide gel electrophoresis (SDS-PAGE), then transferred to nitrocellulose membrane (Sigma) and incubated with specific antibodies. ECL chromogenic substrate was used to visualize the bands and the intensity of the bands was quantified by densitometry (Quantity One software; Bio-Rad, CA, USA). GAPDH was used as a control. Antibodies (1:1,000) for HOXA5, E-cadherin, N-cadherin, vimentin, MMP-2, and MMP-9 were purchased from Cell Signaling Technology (MA, USA).

### Tail vein injection of cells for metastasis in athymic mice

Male athymic mice (5 weeks old) were purchased from the Animal Center of the Chinese Academy of Science (Shanghai, China) and maintained in laminar flow cabinets under specific pathogen-free conditions. SPC-A1 cells transfected with si-HOTAIR or si-NC were harvested from 6-well cell culture plates, washed with PBS, and resuspended at a concentration of 2 × 10^7^ cells/mL. A volume of 0.1 mL of suspended cells was injected into the tail veins of 10 mice. The mice were sacrificed 6 weeks after injection and the lungs were dissected out, photographed and visible tumors on the lung surface were counted. This study was carried out in strict accordance with the recommendations in the Guide for the Care and Use of Laboratory Animals of the National Institutes of Health. The protocol was approved by the Committee on the Ethics of Animal Experiments of Nanjing Medical University (Permit Number: 200933). All surgery was performed under sodium pentobarbital anesthesia, and all efforts were made to minimize suffering [18].

### Statistical analysis

Student’s *t*-test (2-tailed), one-way ANOVA, and the Mann–Whitney U test were conducted to analyze data using SPSS 16.0 software (IBM, IL, USA). *P*-values of less than 0.05 were considered significant.

## Results

### HOTAIR expression is up-regulated in human NSCLC tissues

**Figure 1:**
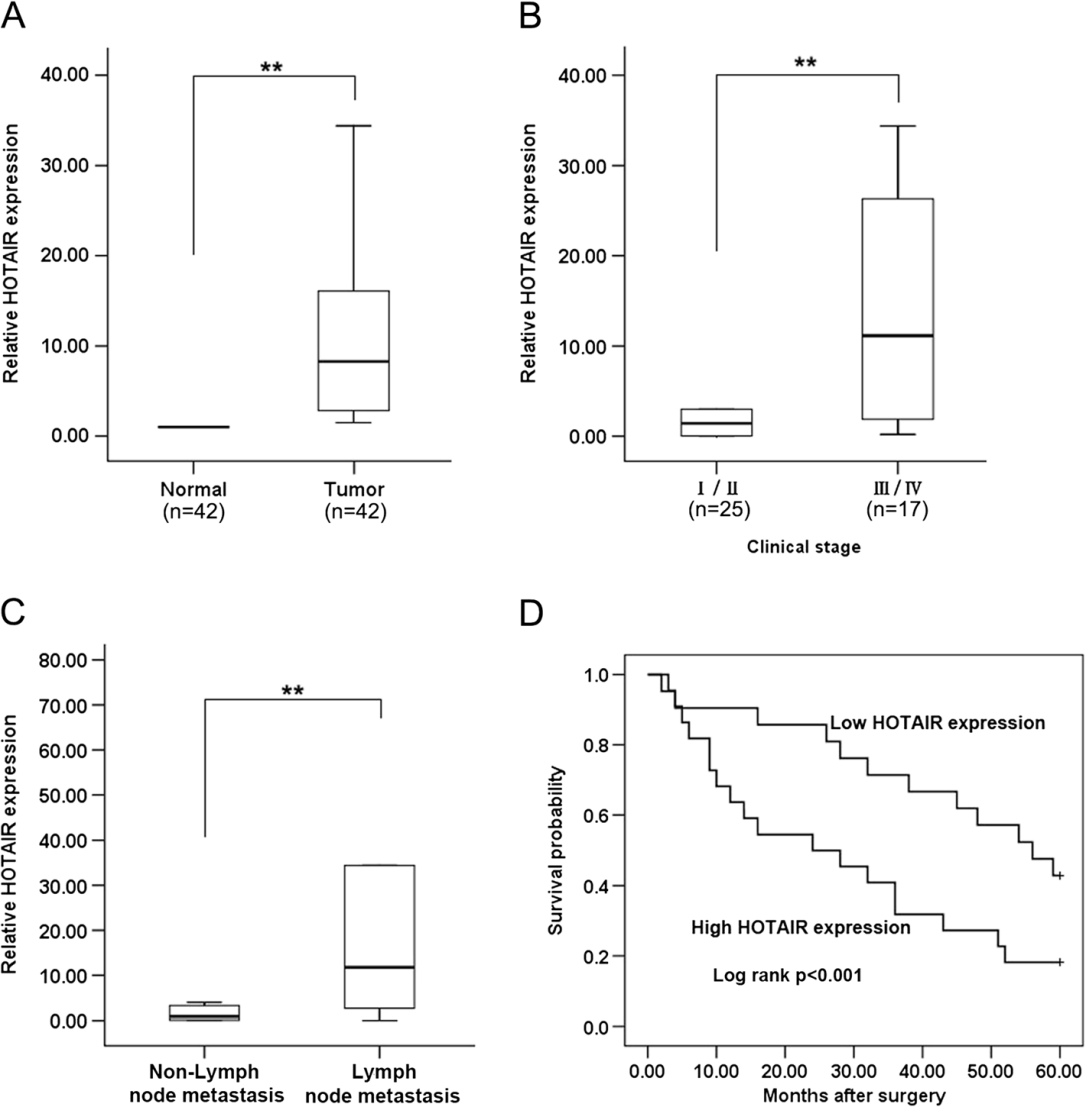
**Relative HOTAIR expression levels in NSCLC tissues and its clinical significance. (A)** HOTAIR was detected in 42 pairs of NSCLC tissues by qRT-PCR. Data are presented as fold change in tumor tissues relative to normal tissues. **(B)** HOTAIR expression was significantly higher in patients at advanced pathological stages. **(C)** HOTAIR expression was significantly higher in patients with lymph node metastasis than in patients with non-lymph node metastasis. **(D)** Patients with high levels of HOTAIR expression showed reduced survival times compared with patients with low levels of HOTAIR expression (*P* <0.001, log-rank test). ** *P* < 0.01.

The level of HOTAIR was detected in 42 NSCLC samples and adjacent, histologically normal tissues by qRT-PCR, and normalized to GAPDH. HOTAIR expression was significantly up-regulated in cancerous tissues compared with corresponding normal tissues (Figure 1A). Furthermore, correlation of HOTAIR expression with pathological features of NSCLC patients, revealed a significant association between HOTAIR up-regulation and advanced pathological stage (I/II, *n* = 25) *vs.* (III/IV, *n* = 17) and NSCLC lymph-node metastasis (Figure 1B and C). However, HOTAIR expression was not associated with patients’ gender or tumor position (Table 1).

**Table 1:** Correlation of the expression of HOTAIR with clinicopathologic features

**Clinicopathologic features**	**n (%)**	**Relative expression of HOTAIR**^**a**^	**P-value**^**b**^
Gender			P = 0.783
Male	32 (76)	11.43	
Female	10 (24)	10.21	
Site of tumor			P = 0.856
Left lung	18 (43)	8.23	
Right lung	24 (57)	9.54	
Differentiation			P = 0.238
Poor	24 (57)	12.8	
High/moderate	18 (43)	7.9	
Lymph node Metastasis			P = 0.005
N0	8 (19)	1.78	
N1	8 (19)	2.59	
N2	10 (24)	10.6	
N3	16 (38)	30.34	

^a^Median of relative expression. ^b^P < 0.05 was considered significant (Mann-Whitney U test between 2 groups and Kruskall-Wallis test for 3 groups).

Kaplan-Meier survival analysis and log-rank tests using patient post-operative survival were performed to further evaluate the correlation between HOTAIR expression and NSCLC patient prognosis. According to the median ratio of relative HOTAIR expression (8.57) in tumor tissues, the 42 NSCLC patients were classified into two groups: High-HOTAIR group (*n* = 21): HOTAIR expression ratio ≥ median ratio; and Low-HOTAIR group (*n* = 21): HOTAIR expression ratio ≤ median ratio. The Kaplan-Meier survival curve showed that patients with high HOTAIR expression levels had significantly shorter survival times compared to those with low HOTAIR expression levels (*P* <0.001; log-rank test) (Figure 1D). These findings support the hypothesis that over-expression of HOTAIR may play a key role in NSCLC progression and development.

### Manipulation of HOTAIR levels in NSCLC cells

**Figure 2:**
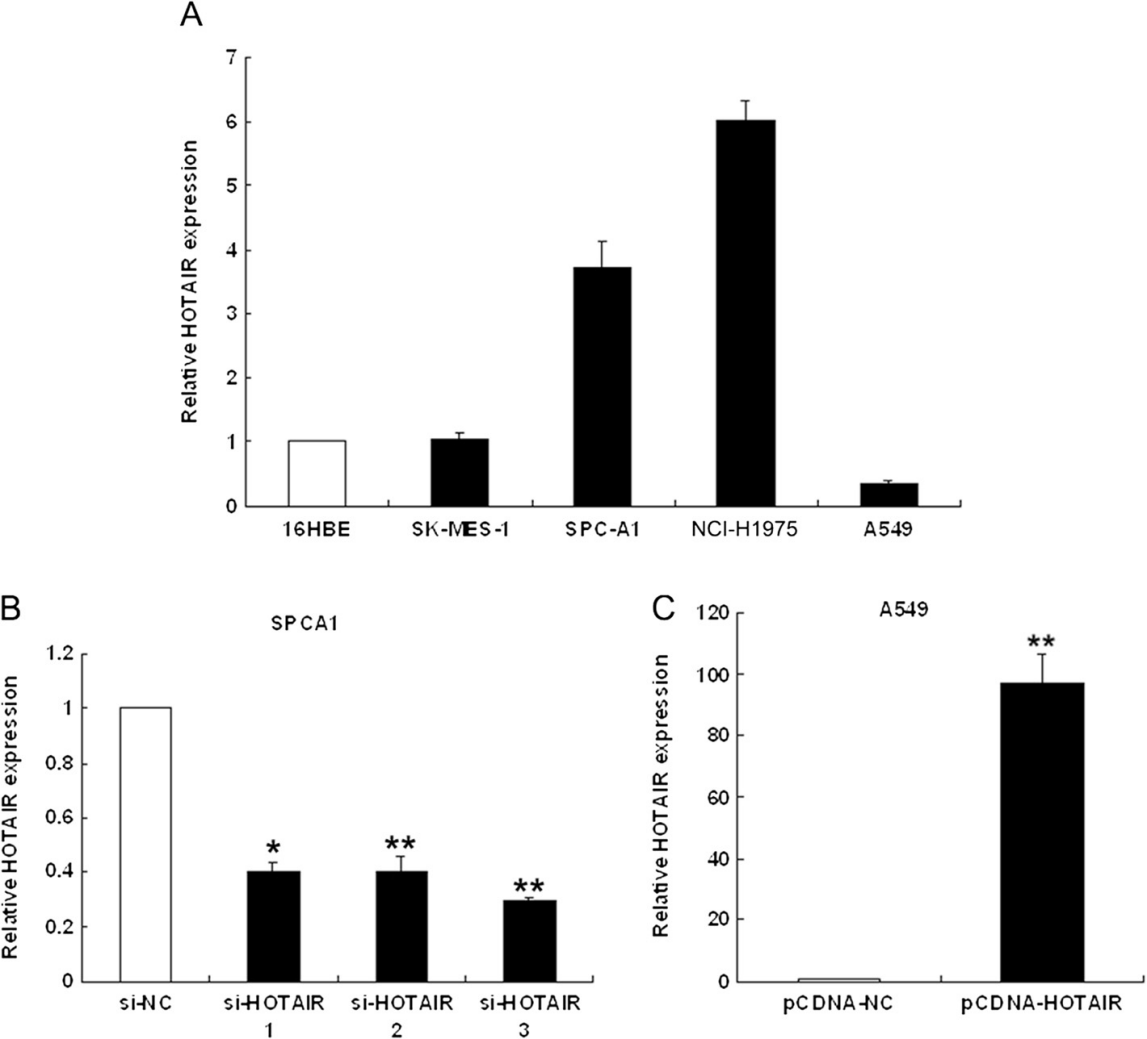
**HOTAIR expression levels in NSCLC cell lines. (A)** qRT-PCR analysis of HOTAIR expression levels in NSCLC cell lines (A549, SPC-A1, NCI-H1650 and SK-MES-1) compared with the normal bronchial epithelial cell line (16HBE). **(B**, **C)** qRT-PCR analysis of HOTAIR expression following treatment of SPC-A1 cells with three individual siRNAs targeting HOTAIR and of A549 cells transfected with pCDNA-HOTAIR. *P < 0.05, **P < 0.01.

We next performed qRT-PCR analysis to examine the expression of HOTAIR in four human NSCLC cell lines, including both adenocarcinoma and squamous carcinoma subtypes. Of these, SPC-A1 and NCI-H1975 expressed significantly higher levels of HOTAIR compared with the normal bronchial epithelial cell line (16HBE), while A549 cells expressed relatively low levels of HOTAIR (Figure 2A).

To investigate the functional effects of HOTAIR in NSCLC cells, we modulated its expression through RNA interference and over-expression experiments. pCDNA3.1-HOTAIR was transfected into A549 cells and three individual HOTAIR siRNAs were transfected into SPC-A1 cells. qRT-PCR analysis of HOTAIR levels was performed 48 h post-transfection. HOTAIR expression was increased 97-fold in A549 cells, compared with control cells. In SPC-A1 cells, when compared with control cells (si-NC), HOTAIR expression was knocked down by 75% by si-HOTAIR3, the most effective siRNA. Thus, si-HOTAIR3 was used in subsequent experiments (Figure 2B and C).

### Effect of HOTAIR on cell proliferation and apoptosis

**Figure 3:**
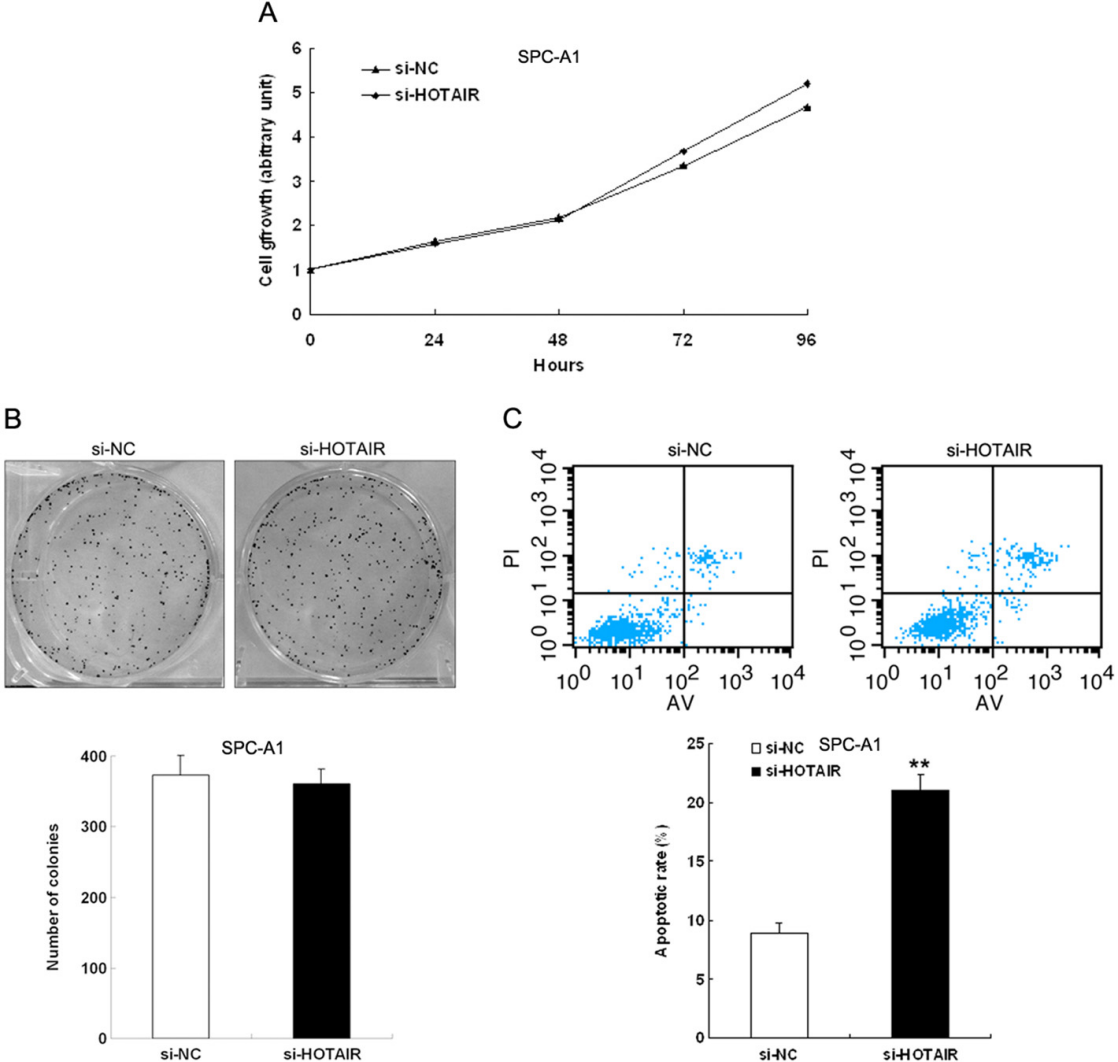
**Effect of HOTAIR on cell proliferation and apoptosis. (A**, **B)** SPC-A1 cells were transfected with si-HOTAIR or si-NC. MTT assays were performed to determine the proliferation of SPC-A1 cells. Data represent the mean ± S.D. from three independent experiments. Colony-forming growth assays were performed, also to determine the proliferation of SPC-A1 cells. The colonies were counted and captured. **(C)** SPC-A1 cells were transfected with si-HOTAIR or si-NC. Apoptotic rates were detected by flow cytometry. UL, necrotic cells; UR, terminal apoptotic cells; LR, early apoptotic cells. **P < 0.01.

To assess the role of HOTAIR in NSCLC, we investigated the effect of targeted knockdown or over-expression of HOTAIR on cell proliferation and apoptosis. MTT assays revealed that cell growth was not influenced in SPC-A1 cells transfected with si-HOTAIR compared with controls (Figure 3A). Similarly, colony-formation assays revealed that clonogenic survival was not changed following inhibition of HOTAIR in SPC-A1 cells (Figure 3B). Consistent with the above results, alteration of HOTAIR expression in A549 cells also had no significant effect on cell proliferation compared with control cells (data not shown).

We then tested the effect of HOTAIR on apoptosis. SPC-A1 cells were seeded in six-well plates and transfected with si-HOTAIR or si-NC. We conducted apoptosis assays using an annexin V-propidium iodide apoptosis detection kit to determine whether knockdown of HOTAIR induces NSCLC cell apoptosis. The results shown in Figure 3C demonstrated a significantly higher percentage of apoptotic cells for si-HOTAIR-treated cells compared to those transfected with the untargeted-control (si-NC). Taken together, these results indicate that inhibition of HOTAIR induces NSCLC cells apoptosis, but has no effect on cell vitality.

### HOTAIR promotes migration and invasion of NSCLC cells

**Figure 4:**
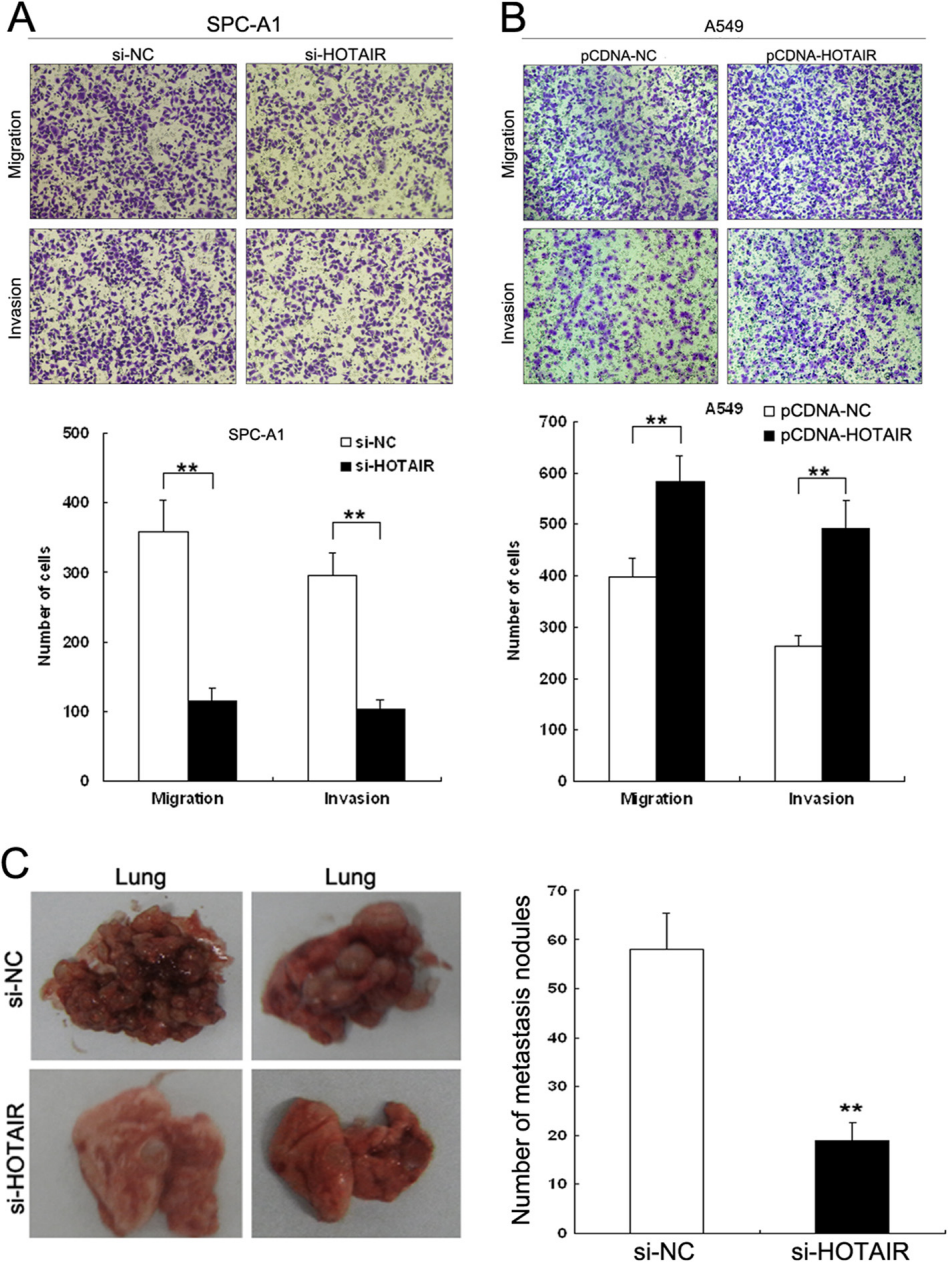
**Effect of HOTAIR on cell migration, invasion and metastasis. (A**, **B)** SPC-A1 cells were transfected with HOTAIR siRNA or si-NC, and A549 cells were transfected with pCDNA-HOTAIR or pCDNA-NC. Transwell assays were performed to investigate the migratory and invasive ability of NSCLC cells. **(C)** Analysis of an experimental metastasis animal model was performed by injecting HOTAIR knockdown SPC-A1 cells into nude mice through the lateral tail vein. Images of lungs from mice in each experimental group are shown. The numbers of tumor nodules on lung surfaces are shown. ** *P* < 0.01.

Cell invasion is a significant aspect of cancer progression, and involves the migration of tumor cells into contiguous tissues and the dissolution of extracellular matrix proteins. To investigate whether HOTAIR has a direct functional role in facilitating NSCLC cell migration and invasion, we evaluated cancer cell invasion through Matrigel and migration through a transwell. As shown in Figure 4A, inhibition of HOTAIR impeded the migration of SPC-A1 cells by approximately 68% compared with control. Similarly, invasion of SPC-A1 cells was reduced by 65% following inhibition of HOTAIR. Conversely, transfection of A549 cells with pCDNA-HOTAIR promoted cell migration and invasion ability ~1.9-fold (Figure 4B). These data indicate that HOTAIR can promote the migratory and invasive phenotype of NSCLC cells.

### HOTAIR promotes NSCLC cell metastasis *in vivo*

To validate the effect of HOTAIR on metastasis of NSCLC cells *in vivo*, SPC-A1 cells transfected with si-HOTAIR were injected into an athymic mouse xenograft model via the tail vein. The metastatic nodules on the surface of lungs were counted after 6 weeks. Inhibition of HOTAIR expression resulted in a significant reduction in the number of metastatic nodules compared with the control group (Figure 4C). This *in vivo* data complemented the functional *in vitro* studies of HOTAIR, and demonstrated that HOTAIR was capable of promoting NSCLC cell metastasis *in vivo*.

### HOTAIR affects the levels of MMP2, MMP9 and HOXA5 proteins

**Figure 5:**
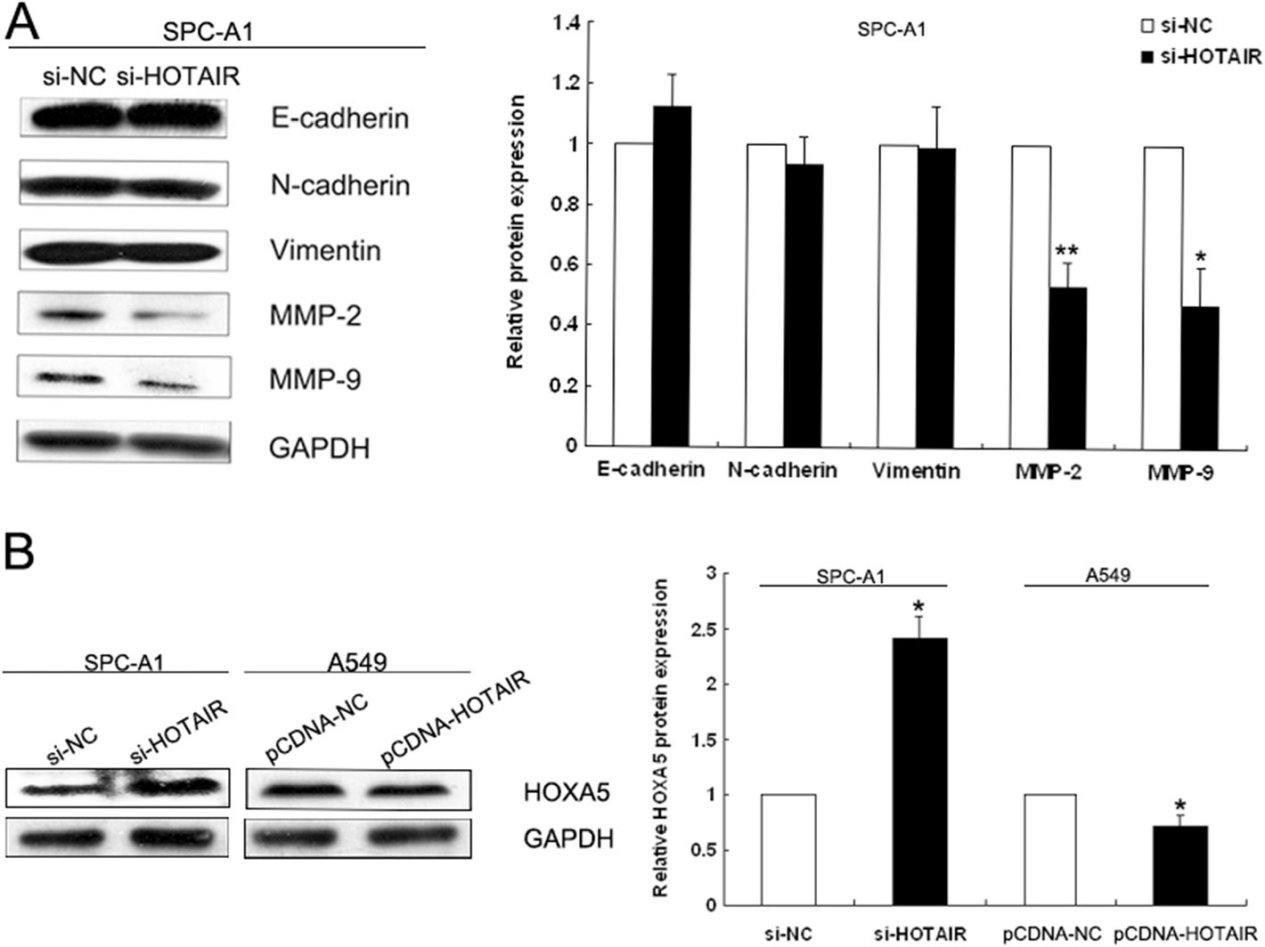
**HOTAIR affects MMP2, MMP9 and HOXA5 protein levels. (A)** Western blot analysis of EMT-induced markers (E-cadherin, N-cadherin and Vimentin) and matrix metalloproteinases (MMP-2 and MMP-9) in HOTAIR knockdown SPC-A1 cells and control cells. **(B)** Western blot analysis of HOXA5 following treatment of SPC-A1 cells with si-HOTAIR and of A549 cells transfected with pCDNA-HOTAIR. GAPDH protein was used as an internal control. * *P* < 0.05; ** *P* < 0.01.

To explore the molecular mechanisms by which HOTAIR contributes to the phenotypes of NSCLC cells, we investigated potential targets involved in tumor invasion and metastasis. Often, cancer cells undergo morphological alterations and change their cell-cell or cell-matrix interactions. The epithelial- mesenchymal transition (EMT) is an important event in the progression of tumor invasion and metastasis. In this study, western blot assays were performed to detect the expression of EMT-induced markers (E-cadherin, N-cadherin and Vimentin) between HOTAIR knockdown SPC-A1 cells and control cells. These results revealed no significant difference between HOTAIR knockdown cells and control cells (Figure 5A). Matrix metalloproteinases (MMPs) have a pivotal role in the degradation of extracellular matrix (ECM) proteins, and thereby enhance the invasive, proliferative and metastatic potential of cancer. Therefore, we next sought to determine whether there was any interaction between MMPs and HOTAIR. As expected, clear reductions in the levels of MMP2 and MMP9 proteins were observed that correlated with the inhibition of HOTAIR in SPC-A1 cells. In addition, suppression of HOTAIR in SPC-A1 cells induced the up-regulation of HOXA5 protein, which is involved in NSCLC cell migration and invasion. Conversely, over-expression of HOTAIR in A549 cells resulted in down-regulation of HOXA5 protein (Figure 5A). These data indicate that HOTAIR may influence the invasive and metastatic potential of NSCLC cells by altering MMPs and HOXA5 expression, but not by deregulating the expression of EMT-induced markers.

## Discussion

NSCLC ranks among the most common and lethal malignant diseases. Poor prognosis of early stage NSCLC is crucially linked to the onset of tumor metastasis [19]. The processes inducing and stimulating metastasis are complex and still not well understood. Schmidt *et al.* have established a role for lncRNA in metastasis formation in NSCLC. They identified the lncRNA, MALAT1 (Metastasis-Associated Lung Adenocarcinoma Transcript 1), as a prognostic marker for metastasis and patient survival in NSCLC [20]. However, the roles of lncRNAs in the carcinogenesis of NSCLC are far from being fully elucidated.

In this paper, we have investigated the involvement of the lncRNA, HOTAIR, in NSCLC carcinogenesis and metastasis. HOTAIR was initially identified as one of 231 lncRNAs associated with the human HOX loci, which, however, can repress transcription *in trans* across 40 kilobases of the HOXD locus in foreskin fibroblasts [21]. Notably, HOTAIR over-expression targets polycomb repressive complex 2 (PRC2), a complex comprised of EZH2, SUZ12 and EED. This has a genome-wide effect, serving to alter H3K27 methylation and gene expression patterns, thus increasing cancer invasiveness and metastasis. Conversely, knockdown of HOTAIR or PRC2 component expression can inhibit cancer invasiveness [15,17]. HOTAIR can also interact with a second histone modification complex, the LSD1/CoREST/REST complex, which coordinates the targeting of PRC2 and LSD1 to chromatin for coupled histone H3K27 methylation and K4 demethylation [22]. Given its important role in the epigenetic regulation of gene expression, it is not surprising that HOTAIR is deregulated in different types of cancer [14-17,23,24]. It remains unclear, however, whether HOTAIR plays an oncogenic role in NSCLC.

The current study indicated that the expression of HOTAIR was dramatically upregulated in NSCLC tissues compared with normal tissues. Specifically, HOTAIR expression was found to be significantly higher at later stages of tumor development and in tumors that had undergone extensive metastasis. Moreover, the overall survival time of patients with lower HOTAIR expression levels was significantly longer than that of patients with higher HOTAIR expression levels. These findings indicate that HOTAIR plays a direct role in the modulation of cancer progression, and may be useful as a novel prognostic or progression marker for NSCLC.

To further assess the role of HOTAIR in NSCLC, we investigated the effects of gain or loss of function of HOTAIR on various aspects of NSCLC biology. First, we demonstrated that RNAi-mediated suppression of HOTAIR in SPC-A1 cells led to a significant inhibition of migration and invasion, and to the promotion of apoptosis. Conversely, introducing HOTAIR into A549 cells, which express relatively low levels of endogenous HOTAIR, induced malignant tumor cell behaviors. To further quantify metastatic potential *in vivo*, we performed tail vein xenografts and compared the rates of lung colonization. The inhibition of HOTAIR expression resulted in a significant reduction in the number of lung metastatic nodules. In conclusion, HOTAIR knockdown can inhibit the invasion and metastasis of NSCLC *in vitro* and *in vivo*; thus, HOTAIR represents a new prognosis marker and a promising target for NSCLC treatment.

To explore the molecular mechanism by which HOTAIR contributes to the invasion and metastasis of NSCLC, we investigated potential target proteins involved in cell motility and matrix invasion. Firstly, a hallmark of EMT is the loss of E-cadherin expression and aberrant expression of N-cadherin and Vimentin [25-28]. Therefore, the protein levels of these EMT-induced markers were investigated after HOTAIR depletion. However, our results indicated that the inhibitory effects on cell migration and invasion were not associated with the epithelial-mesenchymal transition. Metalloproteases (MMPs) are important in many aspects of biology, ranging from cell proliferation, differentiation and remodeling of the extracellular matrix (ECM) to vascularization and cell migration [29]. Here, loss of HOTAIR in NSCLC cells led to a significant decrease in MMP2 and MMP9 protein levels, and the relationship between HOTAIR and MMPs is currently under further investigation in our laboratory.

Of note, HOTAIR can epigenetically regulate HOXD expression, such as HOXD10, by targeting PRC2, leading to H3K27me3 [14,30]. Here, we found that HOTAIR can suppress HOXA5 protein levels, another member of the HOX family. HOXA5 is involved in the developmental regulation of the lung. Mandeville *et al.* observed impaired postnatal lung development in *HoxA5*^*-/-*^ mice, indicating that HOXA5 has a critical role in lung ontogeny, and implying an involvement in lung maturation and function [31]. Similarly, Packer *et al.* reported that HOXA5 is likely to be involved in the development and patterning of the mouse lung [32]. Moreover, dysregulation of HOXA5 expression has been associated with lung tumorigenesis and other diseases in humans [33-35]. In our previously study, we found that HOXA5 was significantly downregulated in NSCLC tissues and inhibition of *HOXA5* expression in A549 cells significantly promotes cell migration and invasion [36]. Consistent with the above findings, ectopic HOTAIR expression in A549 cells also induced corresponding malignant tumor cell behaviors. Taken together, these results indicate that the oncogenic functions of HOTAIR may be partially exerted through its affect on the expression of HOXA5; however, further experiments are needed to elucidate the precise molecular mechanisms by which HOTAIR regulates HOXA5.

## Conclusions

In summary, HOTAIR upregulation may be a negative prognostic factor for NSCLC patients, indicative of poor survival rates and higher risk for cancer metastasis. The current study showed that HOTAIR may regulate the invasion ability of NSCLC cells, partially through regulation of HOXA5 expression. These findings further the understanding of NSCLC pathogenesis and development, and facilitate the development of lncRNA-directed diagnostics and therapeutics against this deadly disease.

## Competing interests

The authors declare that they have no competing interests.

## Authors’ contributions

LXH, DW and WZX were involved in the conception and design of the study. LJ was involved in the provision of study material and patients. LXH, LZL and SM performed the data analysis and interpretation. LXH wrote the manuscript. WZX approved the final version. All authors read and approved the final manuscript.

## Pre-publication history

The pre-publication history for this paper can be accessed here:

http://www.biomedcentral.com/1471-2407/13/464/prepub
